# Hospitals and the Poets. VI. Walt Whitman as a Hospital Orderly

**Published:** 1916-03-18

**Authors:** 


### HOSPITALS AND THE POETS.*

### VI. Walt Whitman as a Hospital Orderly.

WALT WHITMAN, like Henley, made his hospital experiences the basis of poetry. Henley expressed in verse the patient's point of view. Whitman described in *Drum-Taps* his experiences as a hospital orderly during the American Civil War. He was a poet who became, for love of the work, an institutional worker, an inspired male nurse, in fact, whose abnormal temperament found its opportunity in nursing the wounded young men of both armies. It should be remarked in passing, however, that Whitman's finest individual achievement is the *Song of the Open Road*. None the less, the passages to be quoted from *Drum-Taps* have enough fine things in them to show his quality, for anyone, at least, who does not confuse poetic form with the metrical patterns to which the term has been usually, and ignorantly, limited.

### EMERSON TO THE RESCUE.

Born in 1819 at Long Island, the son of a carpenter, at the age of twelve, in 1831, Walt Whitman drifted for a time into a lawyer's and then a doctor's "office," to use the term which he himself chose. The next few years were occupied with work in a printer's office, and teaching in country schools. The compositor now became a journalist and started the *Long Islander* in 1838. For the next ten years he was at work in New York, Brooklyn, and New Orleans in various editorial capacities. After 1848 he spent two years in travelling through the States. On returning to Brooklyn in 1851 he began to make money as a speculative builder. Not being a typical American, however, to make money was not the main object of his life; his heart was really set on "rounding off" *Leaves of Grass*, a process which ended only with his death. The volume was taking shape at this time, if, indeed, the phrase may be used of a work which was covered with ridicule on its appearance on account of its alleged "formlessness" and chaotic confusion. *Leaves of Grass* actually appeared in 1855, a slender quarto, no less memorable perhaps for the prose preface, which set out his aims as a poet, than for the twelve pieces which embodied his conceptions of the new poetry of his desires. A second edition appeared in 1856, and somewhat shamed the laughers by containing Emerson's famous letter to the author, in which, acknowledging a copy of the book, he wrote: "I am not blind to the worth of the wonderful gift of *Leaves of Grass*. I find it the most extraordinary piece of wit and wisdom that America has yet contributed. . . . I find incomparable things, said incomparably well, as they must be." Thoreau's remark, "He *is* Democracy," and President Lincoln's, "Well, he *is* a man," were made about this time. The public, however, were furious, and Whitman's publishers became so frightened that they declined to sell his book. Four years later Whitman produced yet another edition.

### HIS NURSING AT THE FRONT.

The next event in his life occurred in 1862, when the Secession War drew him to the hospitals, first to nurse his brother, and afterwards to remain as a nurse to all. The record of his experience is told in *Specimen Days*, a prose work, which we must pass by without quotation, fascinating though much of it is. The poems suggested by his hospital life during the war are contained in *Drum Taps*, which was first published in 1865.

It was in December 1862, in the first Fredericksburg battle, that Walt Whitman's brother George, who had volunteered in the Northern Army, was wounded. Walt hastened to the camp at Rappahannock, and, after nursing his brother till he was out of danger, went to Washington in charge of a group of wounded Brooklyn soldiers. There he remained as what we should now call an orderly in the military hospitals, the very strenuous work in the wards being diversified by visits to the battlefields. Even his magnificent constitution broke down under the strain. He had a severe illness in 1864, but returned to his work at Washington. This premature return was probably the cause of the paralysis which prostrated him in 1873, and from which, till his death in 1892, he never really recovered. One fruit of his illness was the *Diary of an Invalid*, described by J. A. Symonds as "the brightest and halest" such "Diary" ever written. It is, however, with *Drum Taps*, and his work in the military hospitals, that we are here concerned. In *Not Youth Pertains to Me*, a poem called, as was customary with him, by its opening phrase, Whitman writes:

> I have nourish'd the wounded, and sooth'd many a dying soldier,
> And at intervals, waiting, or in the midst of camp,
> Composed these songs.

As a kind of introduction, *Drum Taps* opens with these lines:

> Aroused and angry
> I thought to beat the alarum, and urge relentless war;
> But soon my fingers fail'd me, my face droop'd, and I resign'd myself,
> To sit by the wounded and soothe them, or silently watch the dead.

In his "prelude," describing the first excitement and enthusiasm for the war, the flags, the cheering, the escort, the partings, Whitman goes on to mention amid "all the mutter of preparation":

> The hospital service—the lint, bandages, medicines;
> The women volunteering for nurses—the work begun for, in earnest—no mere parade now;
> War! an arm'd race is advancing!—the welcome for battle—no turning away;
> War! be it weeks, months, or years—an arm'd race is advancing to welcome it.

* Previous articles in this series appeared on Jan. 8, p. 329; Jan. 22, p. 369; Feb. 5, p. 409; Feb. 19, p. 453; and March 4, p. 499.

The difficulty offered to the reader by these quotations is directly due to their brevity. Whitman, more, I think, than any other poet, demands to be read at large. He makes his appeal to our imaginations by the cumulative pictures which he builds up. Who could appreciate the tumbling cloudlands of a March or a February sky from one feathery plume or boss of vapour? Whitman's poetry is like a cloudland. Cumulus, light and shadow, and rifts of sun, with a headlong wind and a sense of great spaces: these are the broad effects of his verse. The lilt and rhythm of his best passages come home to us only as one unwieldy line follows another as in the ordered disorder of waves breaking on a beach. In this way he gives the effect of open air, wind, or the height of summer in his nature poems; and by similar means, detail following detail, sometimes even in a regular catalogue of occupations, he presents also to our minds the crowded pageant of city life.

### The Retreat from Mons.

Limited, as we are here, to his hospital experiences, we must be content with one relatively long passage, which may startle the memories of some readers who shared in the retreat from Mons:

A march in the ranks hard-prest, and the road unknown;
A route through a heavy wood, with muffled steps in the darkness;
Our army foil'd with loss severe, and the sullen remnant retreating;
Till after midnight glimmer upon us, the lights of a dim-lighted building;
We come to an open space in the woods, and halt by the dim-lighted building;
'Tis a large old church at the crossing roads—'tis now an impromptu hospital;
—Entering but for a minute, I see a sight beyond all the pictures and poems ever made:
Shadows of deepest, deepest black, just lit by moving candles and lamps,
And by one great pitchy torch, stationary, with wild red flame, and clouds of smoke;
By these, crowds, groups of forms, vaguely I see, on the floor, some in the pews laid down;
At my feet more distinctly, a soldier, a mere lad, in danger of bleeding to death, (he is shot in the abdomen;)
I staunch the blood temporarily, (the youngster's face is white as a lily;)
Then before I depart I sweep my eyes o'er the scene, fain to absorb it all;
Faces, varieties, postures beyond description, most in obscurity, some of them dead;
Surgeons operating, attendants holding lights, the smell of ether, the odor of blood;
The crowd, O the crowd of the bloody forms of soldiers —the yard outside also fill'd;
Some on the bare ground, some on planks or stretchers, some in the death-spasm sweating;
An occasional scream or cry, the doctor's shouted orders or calls;
The glisten of the little steel instruments catching the glint of the torches;
These I resume as I chant——

A few more lines would finish the poem, but there is no room for more. If the artistic quality is marred by breaking off here, the actuality of the poem, at any rate, does not suffer. It might have been written at any hour on any day since the march from Mons eighteen months ago! The fact that it remains unique is sufficient proof of its original power. Were this form of verse easy to write—and to suppose so is the first mistake a fool makes about it—somebody else would write it after having been shown the way. As it is, Whitman is unfortunately the undisputed master of this free form—if we except Blake, whose prophetic books (so different in all other respects!) resemble *Leaves of Grass* in this one.

### The Hospital Hero.

From a long poem called "The Dresser," space or no space, room must be found for the following:—

I dress the perforated shoulder, the foot with the bullet wound,
Cleanse the one with a gnawing and putrid gangrene, so sickening, so offensive,
While the attendant stands behind aside me, holding the tray and pail.
I am faithful, I do not give out;
The fractur'd thigh, the knee, the wound in the abdomen,
These and more I dress with impassive hand—(yet deep in my breast a fire, a burning flame).

It was this service to the wounded of both armies which led the public to change their view of him. The uncouth, coarse, ridiculous "New York loafer" became canonised as the "good gray poet." It is difficult to know which phrase is the more objectionable: the abuse or the sentimentality. At all events, the war completed his knowledge of everyday men and women: the mass, the crowd, which was always the "divine average" to him. In the office, on the roads and rivers, and now on the battlefields and in the hospital, he saw the men and women of America "at large." He thus learnt to feel what the framers of a much-abused phrase had once felt when they first inscribed their triple watchword on the banners of the armies of the Revolution. Liberty, Equality, and Fraternity were no catchwords, but living and burning realities to him. He earned the right to say to the people of the United States:—

And now to conceive, and show to the world, what your children *en-masse* really are;
(For who except myself has yet conceiv'd what your children *en-masse* really are?)

This knowledge, the result of personal contact with Nature and men in his many trades and travels, atones for his blatancy, his want of humour, and his feminine vanity (from the masculine type he was singularly free). It saved him, and for his faults, as Meredith said of his frankness of speech, "the nymphs blush, not he." For the world to-day he has this very apposite question:—

Were you looking to be held together by the lawyers?
Or by an agreement on a paper? or by arms?
—Nay—nor the world, nor any living thing, will so cohere.

So far he is the greatest poet who has written convincingly either on hospital life or modern war.

